# Establishment of a recombinase polymerase amplification detection method for *Puccinia striiformis* f. sp. *tritici*

**DOI:** 10.1038/s41598-023-42663-4

**Published:** 2023-09-26

**Authors:** Yaoxia Liu, Jianyun Hao, Qingyun Guo, Jiahui Yan, Qiang Yao

**Affiliations:** Qinghai Provincial Key Laboratory of Agricultural Integrated Pest ManagementScientific Observing and Experimental Station of Crop Pest in Xining, Ministry of Agriculture, Academy of Agriculture and Forestry Science, Qinghai University, Xining, 810016 Qinghai People’s Republic of China

**Keywords:** Field trials, Fungal pathogenesis

## Abstract

Wheat stripe rust caused by *Puccinia striiformis* f. sp. *tritici* (*Pst*) is an airborne disease that endangers wheat during its entire growth period. In this study, the *Pst*134EA_003354 uncharacterized protein (GenBank: XM_047941824.1) of *Pst* was used as the target sequence, and the primers PS-RPA-F and PS-RPA-R, as well as the probe PS-LF-probe, were designed for recombinase polymerase amplification (RPA) technology. Flow chromatography was combined with the process to establish an RPA detection method for *Pst*. This method successfully established visual detection within 10 min under a constant temperature of 39 °C, and the detection results were consistent with those of ordinary PCR analysis. However, it only had high specificity for *Pst*, and the detection limit was 10 fg/μL. In addition, this rapid method successfully detected *Pst* from wheat leaves during the field incubation period, indicating substantial benefits for applied use. In summary, the RPA detection method established in this study has the favourable characteristics of high efficiency, simple functionality, and rapid and universal practicability, providing a theoretical basis for the early detection and prevention of *Pst*.

## Introduction

Wheat (*Triticum aestivum* L.) is the second-largest food crop in China and is one of the most important cereals grown in arid and semiarid regions. It provides 20% of the total calorie and protein intake of the human diet. Wheat stripe rust caused by *Puccinia striiformis* f. sp*. tritici* is one of the most destructive diseases in wheat, causing severe yield loss worldwide^1^. The mass occurrence of wheat diseases caused by phytopathogens is a critical factor that can negatively impact wheat yield^2,3^. In recent years, wheat stripe rust caused by *Puccinia striiformis* f. sp*. tritici* (*Pst*) has caused significant damage in wheat production. Wheat stripe rust is a disease that can occur in plants from the seedling stage to maturity, as *Pst* urediospores infect wheat leaves and undermine their ability to photosynthesize normally, which in turn affects wheat yield^4^. The production of teliospores can occur at all wheat growth stages, but especially when plants reach maturity^5^. Pathogens in plant tissues can be detected using many methods. Plant pathogens causing crop diseases require accurate and rapid detection^6^. Accurate and rapid detection of pathogens is important for the effective management of plant pathogens^7^. Currently, the detection of *Pst* is mainly accomplished by real-time quantitative fluorescent PCR^8^, LAMP detection^9^, molecular labelling methods^10,11^, or ordinary PCR detection methods. However, these technologies impose stringent requirements on personnel and equipment, place high demands on laboratories and are typically not conducive for on-site detection of plant pathogens in environmental or agricultural settings. A rapid and straightforward procedure for detecting *Pst* has traditionally been challenging to develop. RPA, which was invented in 2006, has been experiencing rapid development^12^ and has become a novel isothermal DNA amplification and detection technique^13^.

Recombinase polymerase amplification (RPA) offers a rapid and particular isothermal alternative to PCR^14^. The whole RPA process is very fast. Generally, detectable amplification products can be obtained in approximately 20 min^15^. The technology mainly uses recombinase, single-stranded binding protein, and strand displacement polymerase to specifically recognize and amplify the template at any constant temperature within the range of 25–43 °C. The final target product can be visualized on RPA (lateral flow dipstick, LFD) by adding specific probes^16–18^. Since RPA technology has a fast amplification speed, strong specificity, and high sensitivity, the LFD-RPA product after constant temperature amplification is added to the quantitative buffer solution and reacted with the side stream paper chromatography test strip for 3–5 min, and the test results can be observed and judged by the naked eye. Nucleic acid amplification can be completed by maintaining the activity of the enzyme within a suitable temperature range of 37–42 °C, which does not require high-temperature denaturation, annealing, or other steps. The mild reaction conditions and high amplification efficiency of RPA make it very suitable for rapid clinical diagnosis, food detection, epidemic prevention and control, industrial application, and on-site real-time detection^19^. RPA is gaining popularity because of its unique characteristics, including a low reaction temperature for amplification and a lack of sensitivity to plant inhibitors^20^. Because RPA does not require any advanced laboratory equipment, it is very suitable for on-site testing^21^. At present, RPA technology has not been widely used mainly because it is not a widely available technology and is only used for scientific research^15^. It is important to perform more assessments in the field on isothermal amplification technologies, including LAMP and *RPA* techniques^22^. Furthermore, RPA is more tolerant than PCR to inhibitors and background DNA^23^. Most components in RPA are supplied by the manufacturer in a freeze-dried pellet that allows components to be taken on site without refrigeration^24^. Hence, compared to previously reported methods, the advantages of the RPA method are obvious^25^. It has been used to detect citrus yellow pulse virus, broad bean virus, citrus leaf spot virus, wheat blight, and wheat sheath rot caused by various pathogens^26–30^. However, the detection of *Pst* with RPA has not yet been reported.

In this experiment, a wheat stripe rust *Pst*134EA_003354 uncharacterized protein was used as the target sequence, specific RPA primers and probes were designed and combined with lateral flow layer test strips, and an RPA-LFD rapid detection system for *Pst* was established. No specialized software has been developed for the design of RPA primers, and only PCR software can be used for design and screening^19^. This system was used to detect wheat stripe rust in the field and provides a theoretical basis for the early prevention of *Pst*. The abovementioned nucleic acid detection of *Pst* has achieved good results, which demonstrates that the detection efficiency of the RPA method is obviously better than that of the PCR method, and the reaction time is faster, which provides a rapid and accurate detection method for the disease prediction and field investigation of wheat seedlings in the later growth stage and provides a good basis for the prevention and control of *Pst* to reduce the decline in production and to mitigate economic losses.

## Results

### Primer screening and probe design

**Figure 1:**
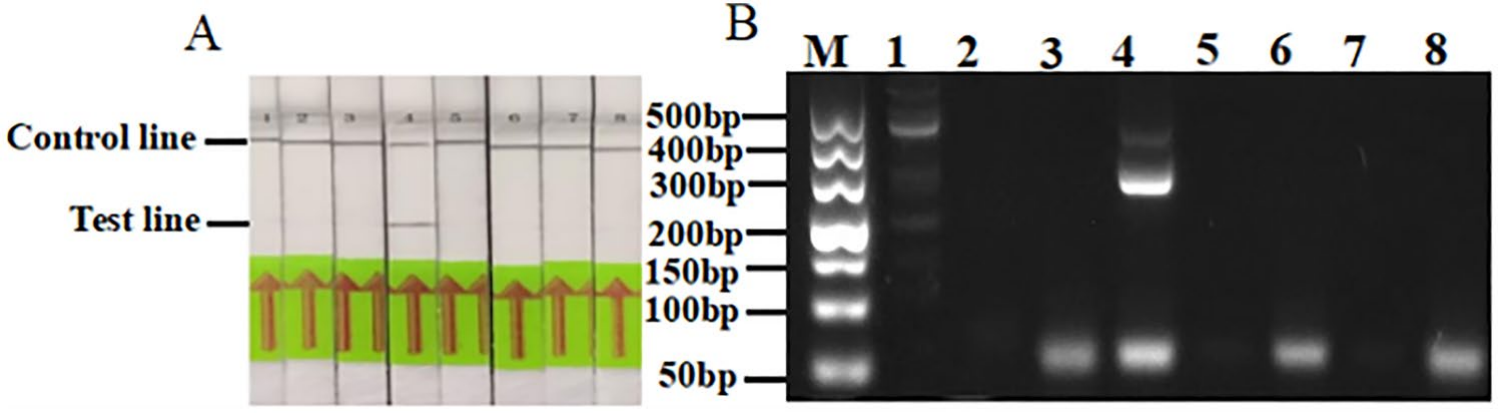
Screening the 8 designed pairs of RPA primers by RPA (**A**) and PCR (**B**). 1: PS1, 2: PS2, 3: PS3, 4: PS4, 5: PS5, 6: PS6, 7: PS7, 8: PS8, M: marker-500.

The resulting eight primer sets were used for RPA detection and agarose gel electrophoresis. The results showed that RPA PS4 primers produced detection bands, while other primer pairs produced only quality control bands. The electrophoresis gel detection results also showed that the PS4 primer pair could amplify a 300 bp target band, primer PS1 produced multiple nontarget fragments, and primers PS2–3 and PS5–8 produced obvious primer dimers. Therefore, the primer PS4 set was selected as the primer for this study. Additionally, probes were designed using fragments with the lowest homology between the amplified product and the downstream sequence (Fig. 1A,B. Fig. S1 shows the original electrophoretic gels and blots.). The primer pairs and probe information used are shown in Table 1.

**Table 1:** Primer and probe sequences and amplicon.

Primer	Sequence	Amplicon (bp)
PS-RPA-F	ATGGTTTTCCACACTGGGATTGGAGCTTAG	300
PS-RPA-R	Biotin-TGTCTTCTGGATTTCCCTTATGTTGTCGTT	
PS-LF-Probe	FAM-TCGGGTTTGGCGGGAACGGCACGGGGCGTG-THF-CGACCCTCGATTGCC-C3-Spacer	

### Specific detection of *Puccinia striiformis* f. sp*. tritici*

**Figure 2:**
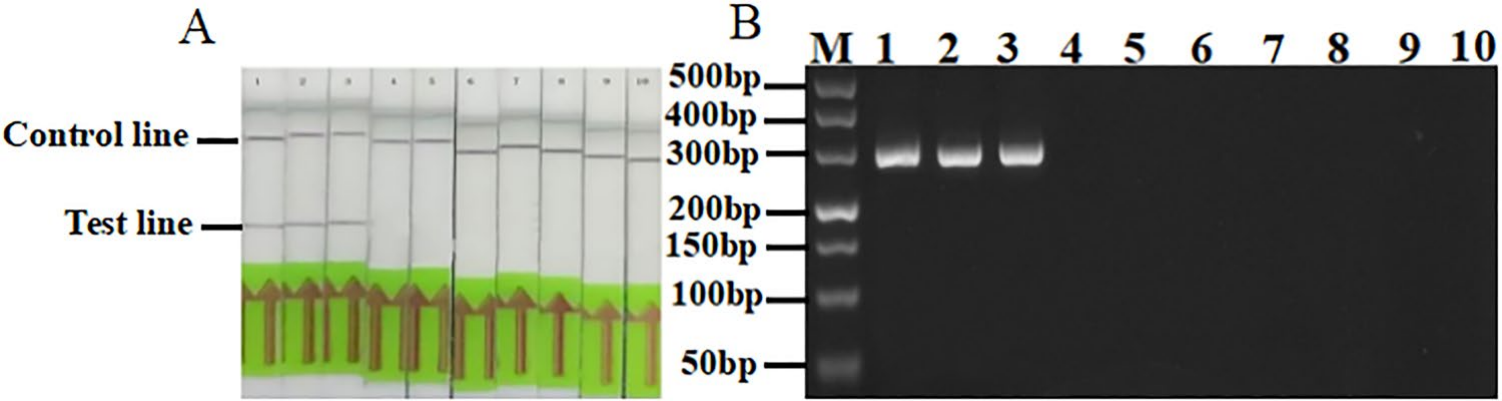
Specific detection of *Puccinia striiformis* f. sp*. tritici* by RPA (**A**) and conventional PCR (**B**). 1. CYR32, 2. CYR33, 3. CYR34, 4. *Puccinia graminis*, 5. *Puccinia recondita*, 6. *Blumeria graminis*, 7. *Puccinia striiformis* West. *f. sp. hordei*, 8. *Puccinia recondita*. f. sp. *agropyri*, 9. wheat leaf gDNA, 10. ddH_2_O, M: marker-500.

The RPA results showed that only the RPA amplification products of *Pst* CYR32, CYR33, and CYR34 DNA showed detection bands and quality control bands on the flow chromatography test strip. Of the five other pathogens tested, only wheat DNA and the blank control showed a quality control zone in ddH_2_O. In addition, PS-RPA-F and PS-RPA-R were primers for ordinary PCR detection, and the agarose gel electrophoresis results showed that *Pst* was amplified to a specific target of 300 bp with no other pathogen amplification occurring. This indicates that the primer had high specificity for *Pst* (Fig. 2A,B. Fig. S2 shows the original electrophoretic gels and blots.).

### RPA sensitivity detection

**Figure 3:**
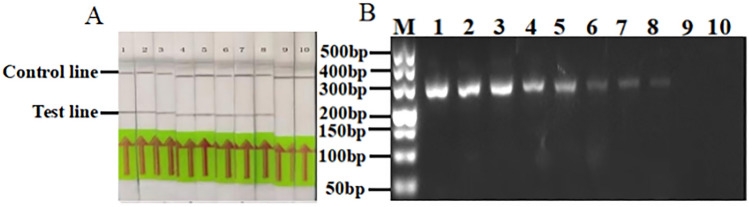
Sensitivity detection of *Puccinia striiformis* f. sp*. tritici* by RPA (**A**) and conventional PCR (**B**). 1–10: 100 ng/μL, 10 ng/μL, 1 ng/μL, 100 pg/μL, 10 pg/μL, 1 pg/μL, 100 fg/μL, 10 fg/μL, 1 fg/μL, 100 ag/μL, M: marker-500.

Following a tenfold dilution of *Pst* gDNA, the flow chromatography test strips showed positive results for 100 ng/μL, 10 ng/μL, 1 ng/μL, 100 pg/μL, 10 pg/μL, 1 pg/μL, 100 fg/μL and 10 fg/μL concentrations, but 1 fg/μL and 100 ag/μL concentrations only produced the quality control band, meaning the result was negative. This illustrated that RPA was successful in detecting as little as 10 fg/μL of *Pst* DNA. The same concentrations of *Pst* gDNA were used for ordinary PCR detection. The results showed that the lower limit of DNA concentration detected by ordinary PCR was also 10 fg/μL. This indicates that the RPA detection system had high sensitivity for *Pst* (Fig. 3A,B. Fig. S3 shows the original electrophoretic gels and blots.).

### Detection of *Puccinia striiformis* f. sp*. tritici* isolates

**Figure 4:**
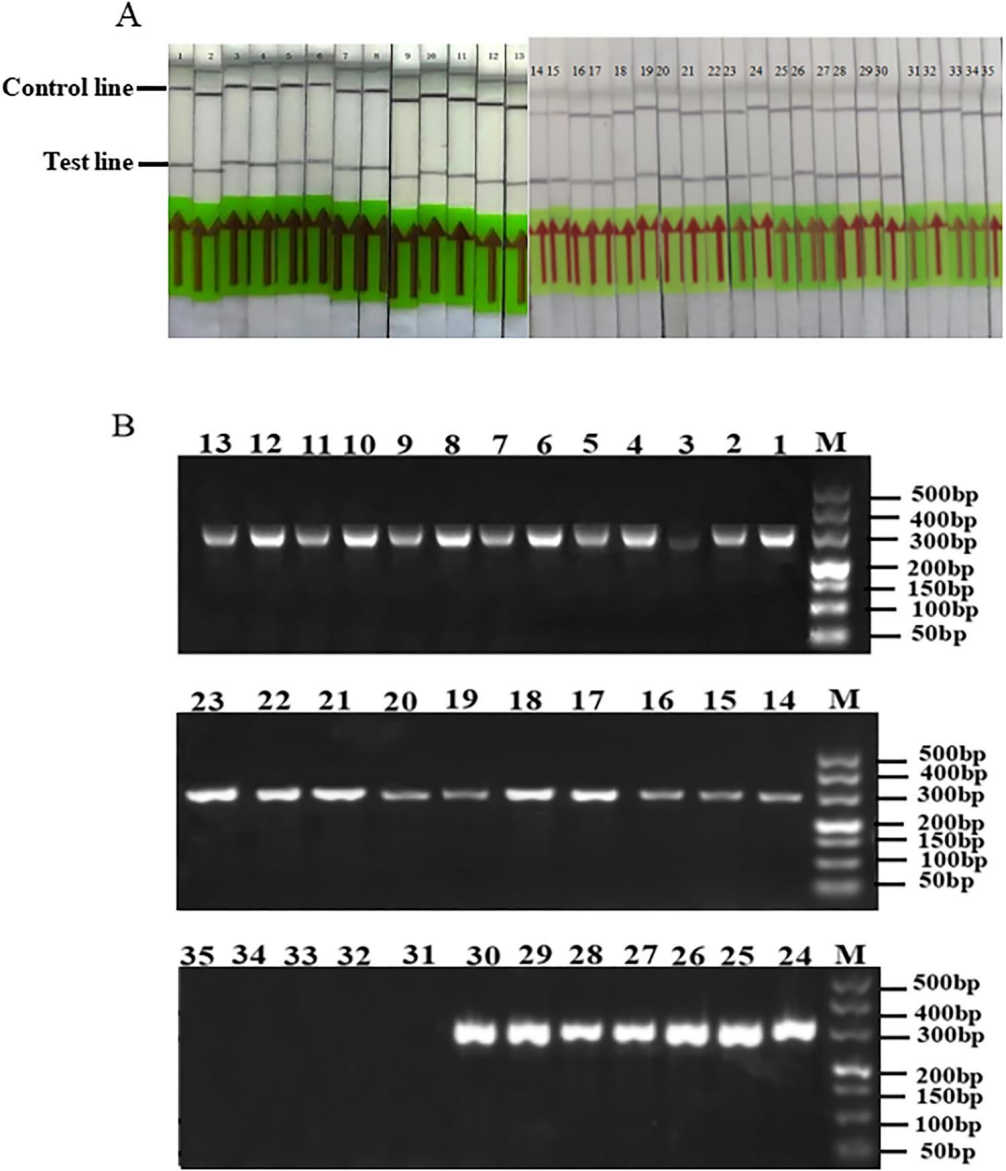
RPA (**A**) and conventional PCR (**B**) detection of *Puccinia striiformis* f. sp*. tritici* isolates*.* 1–30: Different *Puccinia striiformis* f. sp*. tritici.* Sampling points in Datong, Huzhu, Ledu, Jianzha, Hualong and Gui-De Counties, 31–35: uninfected samples, M: marker-500.

Thirty-five samples from Datong, Huzhu, Ledu, Jianzha, Hualong and Gui-De counties were collected. Thirty samples were found to be infected with *Pst* using the RPA test, and five samples were not found to be infected with *Pst*, which was consistent with the results from ordinary PCR analysis. The test results are shown in Fig. 4A,B and Table 2. (Fig. S4 shows the original electrophoretic gels and blots.).

**Table 2:** Sampling points and detection results of isolates of *Puccinia striiformis* f. sp*. tritici.*

Sequence	Area	Location	PCR assay	RPA assay
1	Xining	Kangjia, Datong County	+	+
2		Hanjia, Datong County	+	+
3		Baojia, Datong County	+	+
4		Maojiazhai, Datong County	+	+
5		Benkang, Datong County	+	+
6	Haidong	Gelong, Huzhu County	+	+
7		Mijiazhuang, Huzhu County	+	+
8	Haidong	Xiakou, Ledu County	+	+
9	Huangnan	Yangjia, Jianzha County	+	+
10		Xiaduoba, Jianzha County	+	+
11	Haidong	Xingfu, Hualong County	+	+
12		Longshang, Hualong County	+	+
13	Hainan	Chada, Gui-De County	+	+
14–17	Xining	Kangjia, Datong County	+	+
18–22		Hanjia, Datong County	+	+
23–27		Baojia, Datong County	+	+
28–30		Benkang, Datong County	+	+
31–33	Haidong	Longshang, Hualong County	−	−
34–35	Hainan	Chada, Gui-De County	−	−

“+” experimentally verified positive; “−” experimentally verified negative.

### RPA practicality test

**Figure 5:**
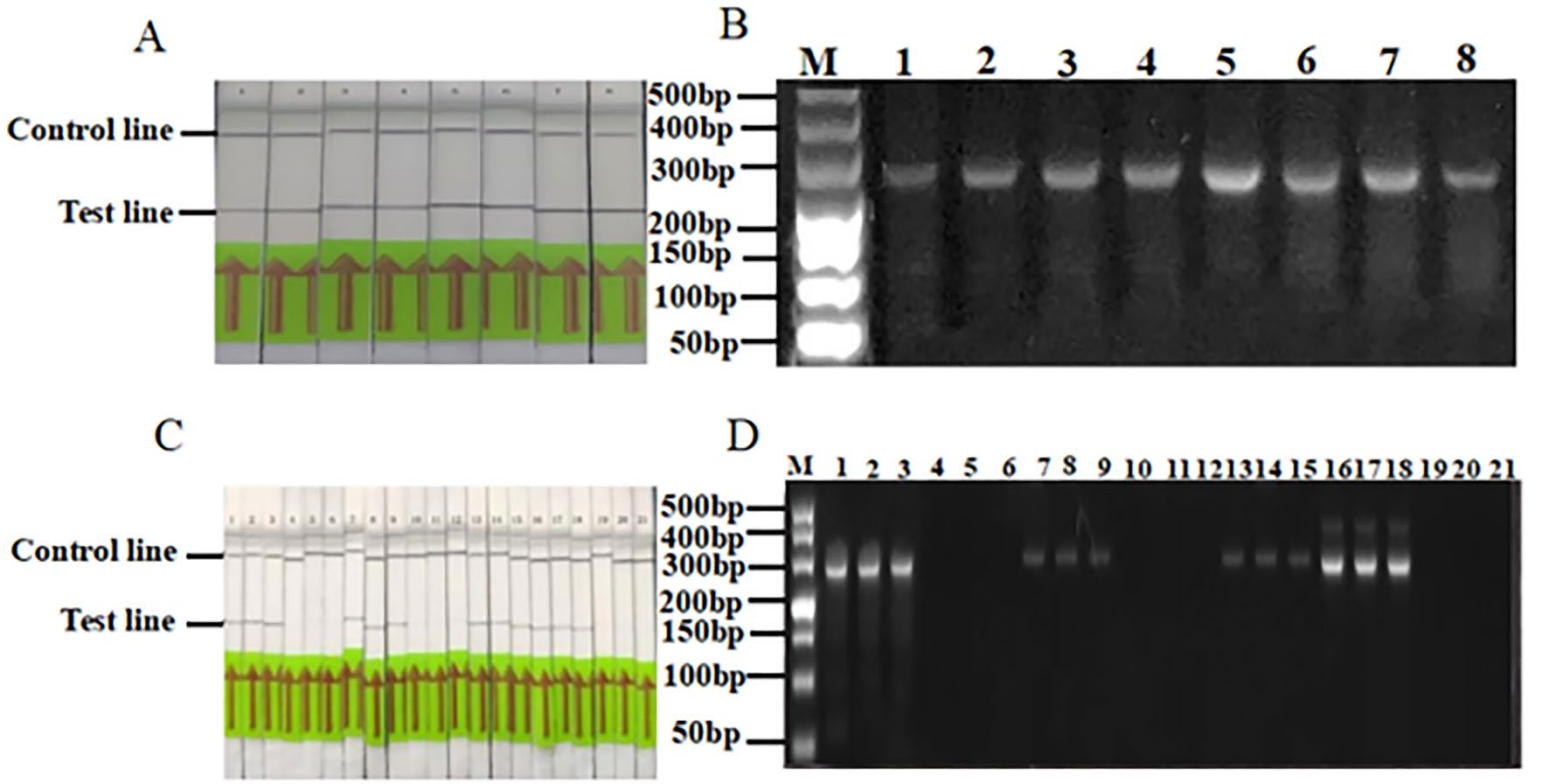
RPA (**A**) and conventional PCR (**B**) detection of *Puccinia striiformis* f. sp*. tritici* isolates after different quantities of days of infection. 1–8: 1 day, 2 days, 3 days, 4 days, 5 days, 6 days, 7 days, 8 days; M: marker-500; Detection of *Puccinia striiformis* f. sp*. tritici* isolates by RPA (**C**) and conventional PCR (**D**). The *Puccinia striiformis* f. sp*. tritici* isolates were collected from 1–3: Dashijia, 4–6: Heerjia, 7–9: Ahetan, 10–12: Xiaduoba, 13–15: Sheren, 16–18: Shangduoba, and 19–21: Baiwujia; M: marker-500.

Twenty-one wheat leaf samples were collected from seven areas in Qinghai Province. The leaves were tested, and the results from the Dashijia, Ahetan, Shangduoba, and Sheren villages were positive. However, *Pst* was not detected in samples from Heerjia, Xiaduoba, and Baiwujia villages. The results from the ordinary PCR tests were comparable. *Pst* was detected on the first day after inoculation. However, the RPA and ordinary PCR analysis showed weak bands, indicating that the amount of *Pst* was relatively low on the first day after infection. *Puccinia striiformis* f. sp*. tritici* was detected from Days 2–8, and observation stopped on the ninth day when sporadic urediospores appeared on the surface of the leaves. This shows that the established RPA system can detect *Pst* at the seedling stage (Fig. 5A–D, and Table 3. Fig. S5 shows the original electrophoretic gels and blots.).

**Table 3:** Application of recombinase polymerase amplification (RPA) in detection isolators of *Puccinia striiformis* f. sp*. tritici* in field.

Sequence	Area	Location	PCR assay	RPA assay
1–3	Hainan	Dashijia, Gui-De County	+	+
4–6		Heerjia, Gui-De County	−	−
7–9	Haidong	Ahetan, Hualong County	+	+
10–12		Xiaduoba, Hualong County	−	−
13–15		Sheren, Hualong County	+	+
16–18		Shangduoba, Hualong County	+	+
19–21		Baiwujia, Minhe County	−	−

“+”experimentally verified positive; “−”experimentally verified negative.

## Discussion

In this study, we designed and screened specific RPA primers and probes based on the *Pst*134EA_003354 uncharacterized protein and established a rapid detection method for *Pst* using RPA technology in combination with lateral flow chromatography test strips to detect *Pst* visually during the incubation period.

Primer and probe design are key factors in the success of RPA detection. The length of RPA primers is generally between 30 and 35 bp; however, TwistDx has recently announced that PCR primers (18 bases and higher) can successfully be used^31^. The target band of the amplified product in this study was between 100 and 500 bp. The primers PS-RPA-F and PS-RPA-R and the probe PS-LF-Probe used in this study specifically detected the physiological races CYR32, CYR33, and CYR34 and can be used to detect the pathogens of other wheat leaf diseases. This illustrates that the specificity of the test was strong. It has been reported that the detection limit of the LAMP detection system for *Pst* is 1 pg/μL^9^, and the dual real-time quantitative fluorescent system established by Pan et al. can detect *Pst* at a minimum of 0.4 pg/μL^32^. When using RPA technology to detect other diseases, the detection sensitivities have been reported as follows: Wei et al. reported a sensitivity of 75 fg/μL for the detection of the causal agent of tomato leaf bacterial spot pathogen^33^, the RPA detection sensitivity of *Pythium aphanidermatum* established by Zhao et al. was 3.75 fg/μL^34^, and Shen constructed an RPA for the detection of tobacco and found the sensitivity of bacterial wilt to be 1 pg/μL^35^. Relatively speaking, the PS-RPA-F, PS-RPA-R, and PS-LF-Probe established in this study had a lower detection limit of 10 fg/μL for *Pst* genomic DNA, and the sensitivity was relatively high.

LAMP detection of *Pst* requires the design of four primers and a loop primer for amplification^36^, while RPA technology requires only forwards and reverse primers to detect the target band. Additionally, the complexity of RPA primer design is relatively low compared to LAMP detection technology, and the RPA technique can specifically detect *Pst* within 20 min at a constant temperature of 39 °C. The method is simple, fast, and can be conducted at room temperature, and the detection buffer can be divided into aliquots to avoid false-positive results. The practicality of RPA technology is an important factor warranting the promotion of this method.

In this study, thirty mixed isolates of *Pst* and five uninfected samples were collected from the Datong, Huzhu, Ledu, Jianzha, Hualong and Gui-DeGui-De counties of Qinghai Province, and 21 self-inoculated leaves of spring wheat seedlings were collected from areas with a high incidence of *Pst* that were infected for 1–8 days. A small amount of *Pst* was found in the mixed isolates on the first day after infection. In addition, 12 samples were detected in areas with a high incidence of *Pst*. This shows that the application of this system can rapidly and efficiently detect *Pst* in leaves during the incubation period. This study provides a theoretical basis for the early prevention and control of wheat stripe rust.

The technique has been applied to detect wheat take-all^32^ and wheat sheath blight^33^. RPA detection is of great significance for the early diagnosis, prediction, and comprehensive control of various wheat diseases. In the future, we will use this technique to distinguish the physiological races of wheat stripe rust. The traditional method is to use differential hosts. This process is long and complex. However, if RPA detection technology is used, the physiological race can be identified quickly, saving time, material resources, and workforce. This will be the direction of our subsequent efforts.

## Materials and methods

### Test materials and reagents

The test pathogens in this study were *Puccinia striiformis* f. sp. *tritici* races CYR32, CYR33, CYR34; isolates of *Pst*; *Puccinia graminis* Pers *tritici* Eriks and E Henn; *Puccinia recondita*, *Blumeria graminis* f. sp. *tritici*, *Puccinia striiformis* West. f. sp., *Puccinia recondita.* f. sp. *agropyri*. The pathogens were provided by the Key Laboratory of Comprehensive Management of Agricultural Pests of Qinghai Province and the College of Plant Protection, Northwest A&F University.

**Figure 6:**
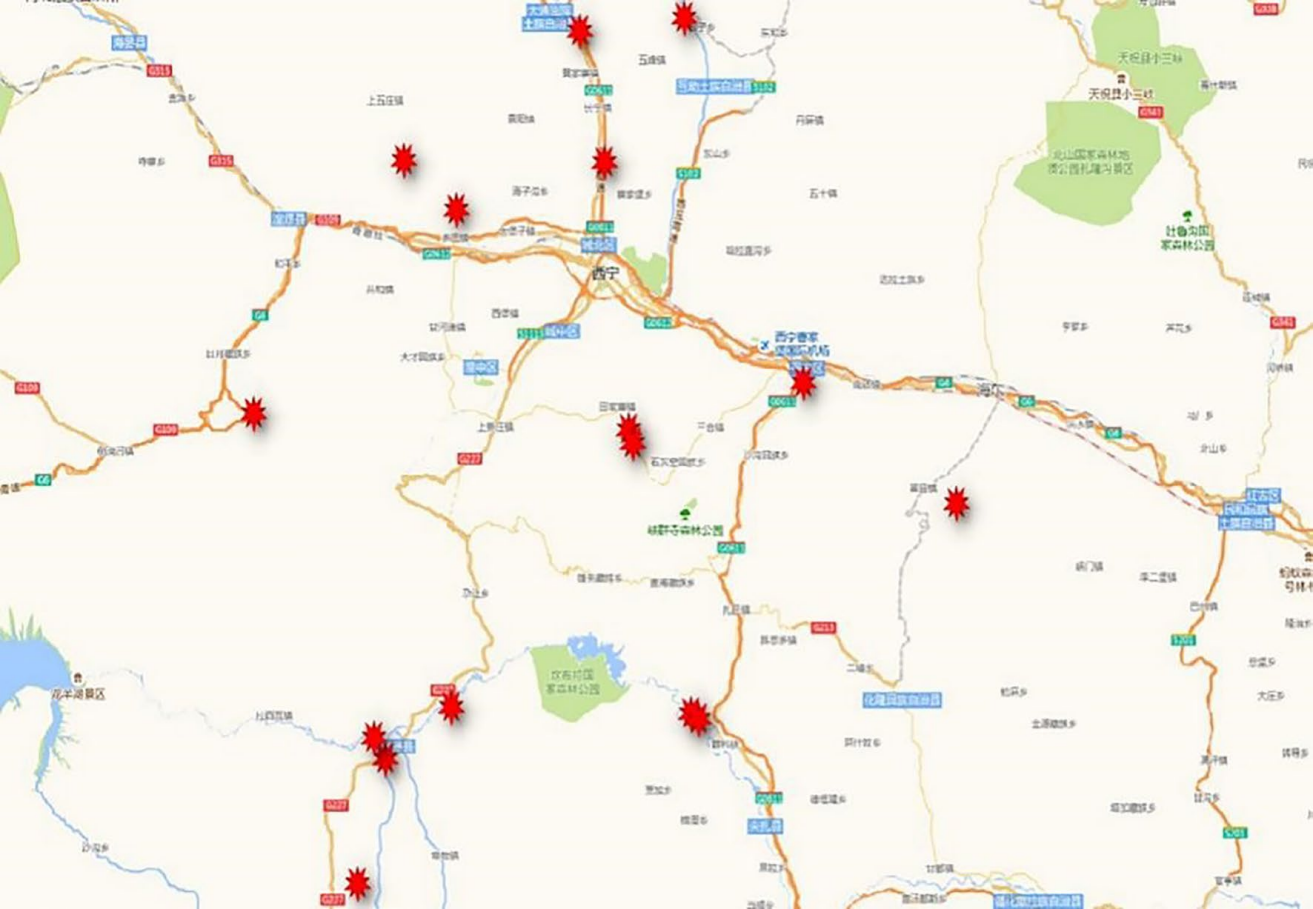
The sampling site marker map of the *Puccinia striiformis* f. sp. *tritici* sample isolates.

The test materials in this study were Mingxian 169 wheat seedling leaves collected from the Dashijia, Heerjia, and Chada villages in Gui-De County; from the Shangduoba, Xiaduoba, Xingfu, and Longshang villages in Hualong County; from Ahetan village in Xunhua County; from Baiwujia village in Minhe County; from the Kangjia, Hanjia, Baojia, Maojiazhai and Benkang villages in Datong County; from Gelong village in Huzhu County; from Xiakou village in Ledu County; and from Jianzha County in Yangjiacun (Fig. 6). There was a distance of at least 50 m between samples in a single wheat field to avoid duplication of any original isolate during the season.

The reagents used in this study were as follows: RAA-nfo nucleic acid amplification reagent (type test strip) was acquired from Qinghai Baisai Trading Co., Ltd.; HybriDetect was purchased from Milenia Biotec Versailler Straße, Gießen Milenia; Premix Ex TaqTM II was purchased from TaKaRa; Shanghai Sangon Biotech Co., Ltd. synthesized all the primers that were used. Ordinary PCR is in vitro enzymatic synthesis of specific DNA fragments, which is based on the level of DNA concentration. In this experiment, the detection band began to weaken when the DNA concentration was lower than 1 pg/μL. The reaction mode of the RPA technology is different from that of PCR in that it does not require thermal cycle steps such as denaturation and annealing. This technology mainly relies on recombinase, polymerase, and single-stranded binding protein (SSB).

### Extraction of pathogenic fungus DNA

Wheat stripe rust samples were stored at 4 °C in a desiccator. There were 10 blades of Mingxian 169 and 30 blades of each sample at each sampling point. The genomic DNA of *Pst* and other control pathogens was extracted using the (Cetyltrimethylammonium Bromide)CTAB method^37^. The concentration of the obtained DNA was measured using a NanoDrop™ One ultramicro ultraviolet spectrophotometer, and the DNA integrity was checked using a 2% agarose gel. It was stored at − 80 °C until use.

### RPA reaction system establishment

Preparation began by adding 40.9 μL of buffer A, 2 μL of forwards and reverse primers (10 μmol/L), and 0.6 μL of probe (10 μmol/L) to the detection tube containing the test dry enzyme preparation. Then, 2.0 μL of the test sample DNA and 2.5 μL of buffer B were added. This was mixed thoroughly by inverting the tube 5–6 times; then, the solution was centrifuged at a low speed for 10 s. The PE tube was then placed in a water bath at a constant temperature of 39 °C and incubated for 10 min. After the reaction, 50 μL DNA extract (volume ratio = 24:25:1) was added for extraction, the tube was centrifuged at 12,000 rpm for 5 min, and the supernatant was collected. Finally, 20–40 μL of the supernatant was diluted 20–50 times with sterile water or PBS, resulting in a dilution volume that was not less than 100 μL. RPA-LFD technology is based on the principle of RPA amplification. The primers with the biotin marker and the probe with the carboxyl fluorescein (FAM) marker are used to amplify the target nucleic acid so that the final amplification product carries both FAM and biotin marker LFD. The front end is coated with gold nanoparticles with FAM antibody, and the detection line is covered with a biotin antibody. When the reaction solution enters the test strip, the amplification product with FAM and biotin forms a biotin antibody-nucleic acid-gold nanoparticle complex on the detection line through antigen–antibody binding and colour development. There is also a quality control line on the LFD, which is coated with a fixed antibody, that can be directly combined with gold nanoparticles with FAM antibody and coloured on the quality control line to ensure the effectiveness of the test strip.

### The ordinary PCR system

A specific primer of *Pst*, PS4F/PS4R, was used for ordinary PCR detection. The reaction system was as follows: MasterMix 12.5 μL, primers (10 μmol/L) 1 μL each, template 1 μL, and ddH_2_O 9.5 μL. The amplification program was as follows: 95 °C predenaturation for 3 min; 35 cycles of 95 °C denaturation for 1 min, 67.2 °C annealing for 30 s, and a 72 °C extension for 1 min; extension at 72 °C for 5 min; and storage at 4 °C. A sample of 4 μL was taken for electrophoresis.

### Design of the RPA primers and probes

In this study, the *Pst*134EA_003354 uncharacterized protein was used as the target sequence (GenBank: XM_047941824.1) in conjunction with the characteristics of RPA primers. The primers and probe were designed using the Twist Amp nfo assay design manual guidelines (http://www.twistdx.co.uk). Eight sets of specific RPA primer pairs for *Pst* were designed based on the *Pst*134EA_003354 uncharacterized protein sequence (GenBank: XM_047941824.1) of *Pst* using Primer Premier 3.0 software (Table 4). The standard parameters of RPA primer design were taken into consideration, and primer specificity was considered using primer-BLAST software (http://www.ncbi.nlm.nih.gov). CYR32 was selected as the source of *Pst.* DNA containing specific fragments of *Pst* was amplified by PCR, and the PCR stock solution of *Pst* was sequenced by amplification. The PCR stock solution was sequenced and analysed utilizing the NCBI database. After initial screening by conventional PCR and RPA, one optimally performing primer set (PS-RPA-F/PS-RPA-R) that consistently amplified an ~ 300 bp specific region was selected. The reverse primer (PS-RPA-R) was conjugated with the antigenic biotin molecule at the 5′ end. DNAMAN V6 software was used to compare the sequences to confirm the accuracy of the products and design the probes. The corresponding TwistAmp nfo probe (PS-LF-Probe) was designed by modifying the 31st nucleotide with a base analogue tetrahydrofuran (THF) residue and the 5ˈ terminus labelled with FAM, whereas the 3′ terminus was designed to contain a C3-spacer polymerase extension blocker. Shanghai Sangon Biological Engineering Co., Ltd. performed the fragment design probe, sequencing, and primer design.

**Table 4:** Recombinase polymerase amplification (RPA) primer sequences.

Primer	Sequence
*PS1F*	TATCCGTGAATGTGGTTATCAGGATGGTTT
*PS1R*	AATTTCCCTTATGTTGTCGTTCGCATAGTA
*PS2F*	TCCGTGAATGTGGTTATCAGGATGGTTTTC
*PS2R*	AATTTCCCTTATGTTGTCGTTCGCATAGTA
*PS3F*	ATGTGGTTATCAGGATGGTTTTCCGCACTG
*PS3R*	AATTTCCCTTATGTTGTCGTTCGCATAGTA
*PS4F*	ATGGTTTTCCACACTGGGATTGGAGCTTAG
*PS4R*	TGTCTTCTGGATTTCCCTTATGTTGTCGTT
*PS5F*	GGTATCCGTGAATGTGGTTATCAGGATGGT
*PS5R*	AATTTCCCTTATGTTGTCGTTCGCATAGTA
*PS6F*	ATGTGGTTATCAGGATGGTTTTCCGCACTG
*PS6R*	TGTCTTCTGAATTTCCCTTATGTTGTCGTT
*PS7F*	CGGTCGCACCTGCCCCAAAATCAACACCAG
*PS7R*	CATCGGCGGAGTCTAAGCTCCAATCCCAGT
*PS8F*	GAATGTGGTTATCAGGATGGTTTTCCGCACTG
*PS8R*	AATTTCCCTTATGTTGTCGTTCGCATAGTA

### RPA-specific detection of *Puccinia striiformis* f. sp*. tritici*

In this study, the physiological races CYR32, CYR33, and CYR34 of *Pst* were selected. These are currently the main common physiological *Pst* species in wheat production in China, and the above physiological races have been identified via differential Chinese hosts of *Pst* that consisted of 19 wheat varieties, with the isolates identified being *Puccinia striiformis* f. sp*. tritici*; *Puccinia graminis* Pers *tritici* Eriks and E Henn; *Puccinia recondita*; *Blumeria graminis* f. sp. *Tritici*; *Puccinia striiformis* West. f. sp.; and *Puccinia recondita.* f. sp. *agropyri*. Wheat gDNA served as the control bacteria, and water was used as the negative control. PS-RPA-F and PS-RPA-R primers and the probe PS-LF-Probe were used to test the specificity of RPA, which was verified by conventional PCR.

### RPA-sensitivity testing of *Puccinia striiformis* f. sp*. tritici*

A 1 μL sample of CYR33 gDNA was taken, and a tenfold serial dilution was conducted to obtain the appropriate concentration as follows: 100 ng/μL, 10 ng/μL, 1 ng/μL, 100 pg/μL, 10 pg/μL, 1 pg/μL, 100 fg/μL, 10 fg/μL, 1 fg/μL, and ultimately, 100 ag/μL. The RPA and an ordinary PCR test were performed, and the results were compared.

### Detection of *Puccinia striiformis* f. sp*. tritici* isolates

Wheat volunteer (self-sown) seedlings and late-autumn seedlings were collected in the Datong, Huzhu, Ledu, Jianzha, Hualong and Gui-De Counties of Qinghai Province. *Pst* had occurred in the standard samples. Obvious *Pst* urediospores appeared on some of the leaves, but the physiological races of the standard samples were not yet determined. Therefore, RPA and PCR analyses were performed to assess the presence of mixed fungi.

### The practicality of the RPA testing system

Mingxian 169 wheat leaves were inoculated with CYR34, and samples were taken after 24 h of humidification culture at 5–10 °C between inoculations. After 1–8 days, leaves were collected to extract DNA. Historically, Gui-De, Xunhua, Hualong, and Minhe counties have frequently suffered from wheat stripe rust outbreaks^38^. The diseased plants were marked in late November 2020, and the marked seedling leaves were collected at the end of March 2021, when no *Pst* uredospores had appeared on the surface of the leaves. Twenty-one leaf samples were collected for DNA extraction and stored at − 80 °C. RPA detection and conventional PCR detection methods were used to detect whether the samples were infected with *Pst*.

### Ethical approval

Our study complies with relevant institutional, national, and international guidelines and legislation. The use of plants in this study, whether cultivated or wild, as well as the collection of plant material, were in line with the policies of the relevant institutions and with national and international norms and laws.

### Supplementary Information

Supplementary Figures.

## Data Availability

All data generated or analysed during this study are included in this published article.
