# Supplementary material for: Establishment of a recombinase polymerase amplification detection method for Puccinia striiformis f. sp. tritici
Source: Sci Rep. 2023 Sep 26;13:16133. doi: 10.1038/s41598-023-42663-4 (PMC10522615; doi:10.1038/s41598-023-42663-4)
Supplement: Supplementary file 1 — Supplementary Figures. [file 41598_2023_42663_MOESM1_ESM.docx]

**
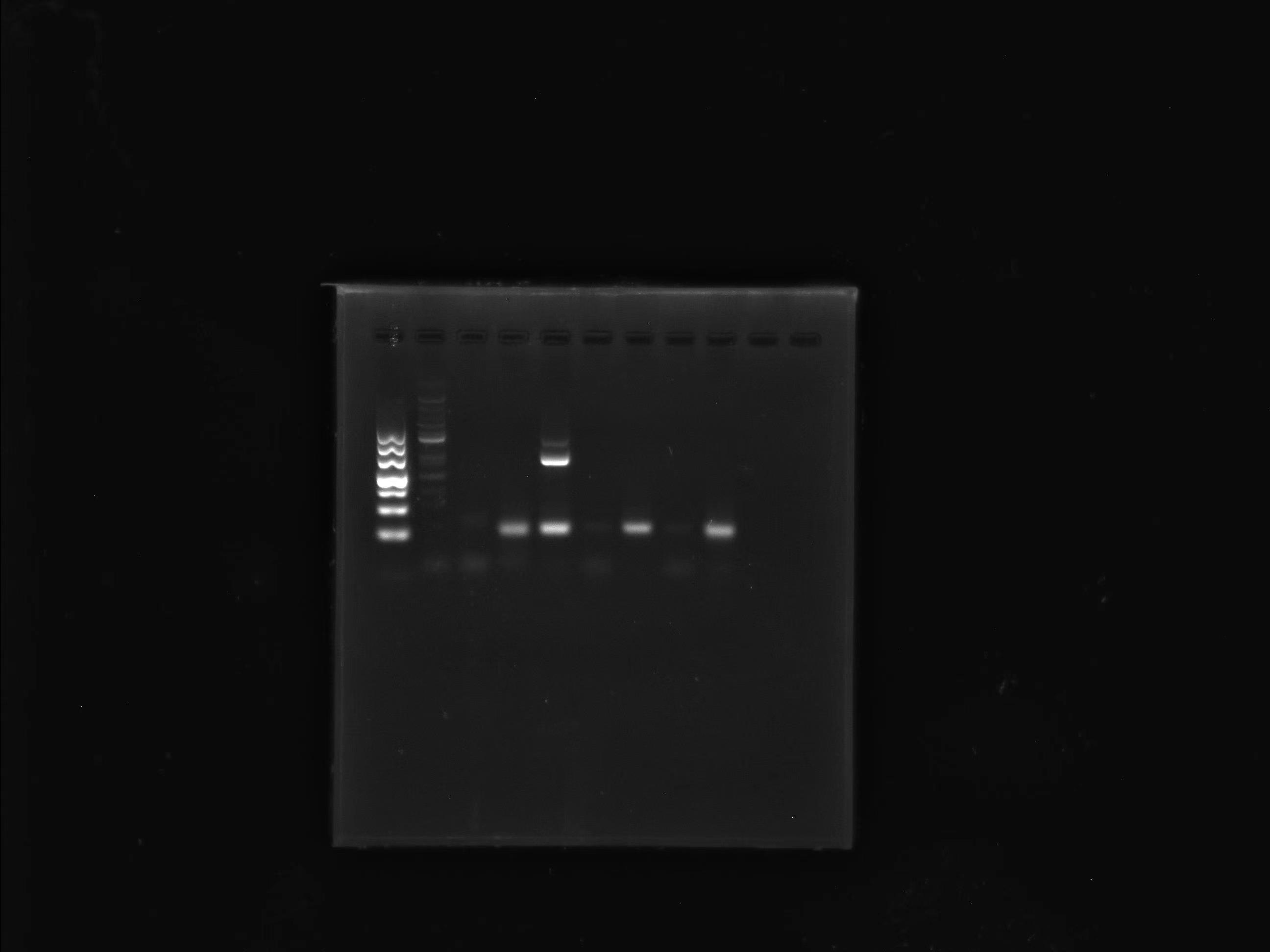
**

**Figure S1.** Screening the designed 8 pairs RPA primers by PCR.


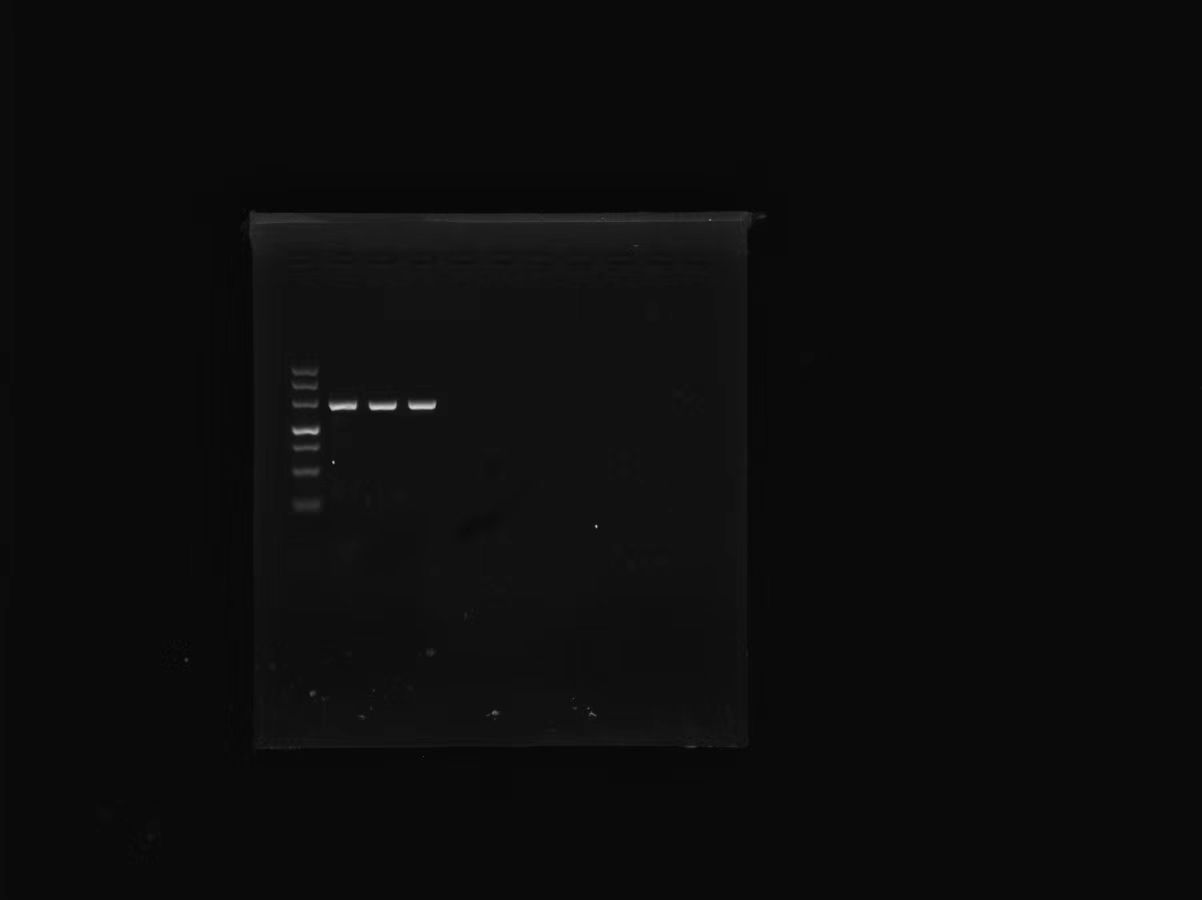


**Figure. S2.** Specific detection of *Puccinia striiformis* f. sp*. tritici* by conventional PCR.


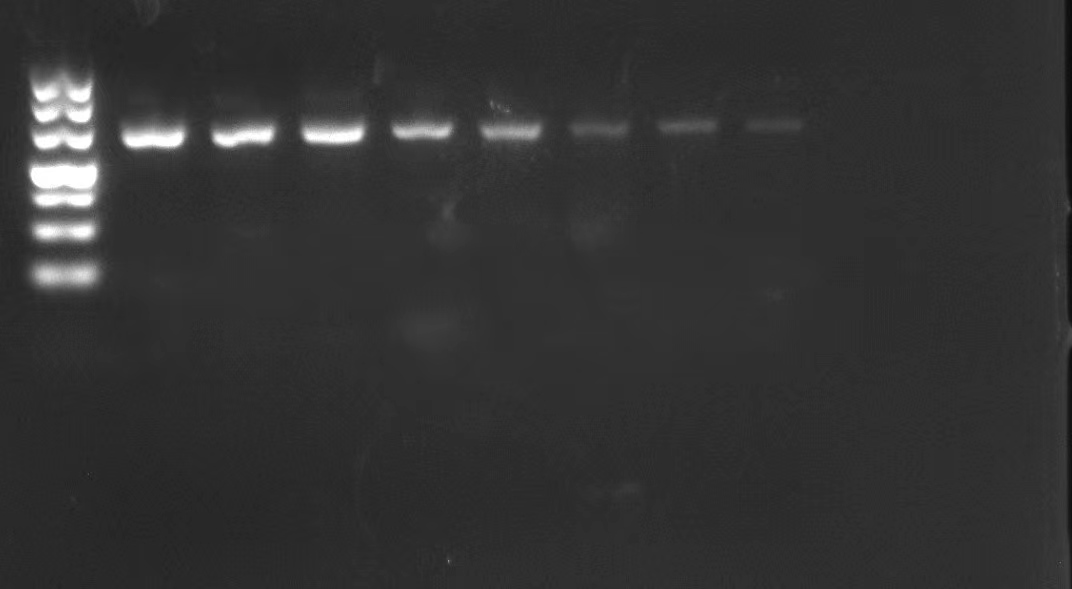


**Figure. S3.** Sensitivity detection of *Puccinia striiformis* f. sp*. tritici* by conventional PCR.


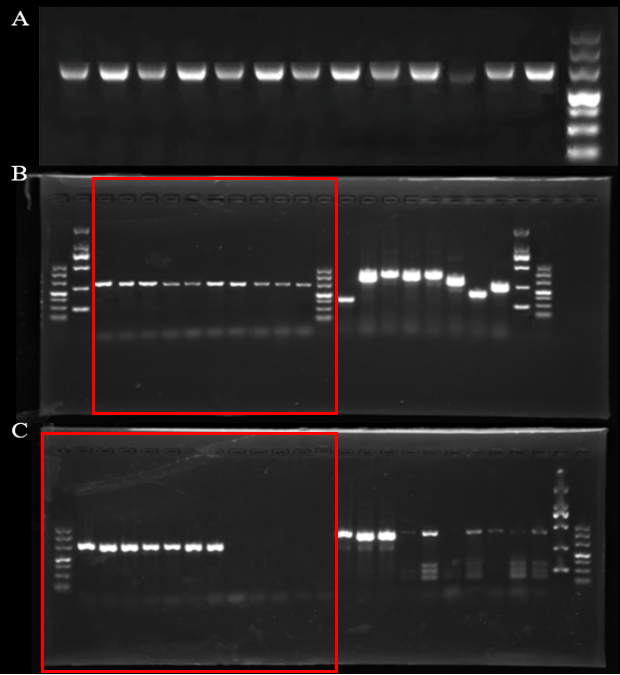


**Figure. S4.** Conventional PCR detection of *Puccinia striiformis* f. sp*. tritici* isolates*.* The red box in the figure shows the experimental results in the article.


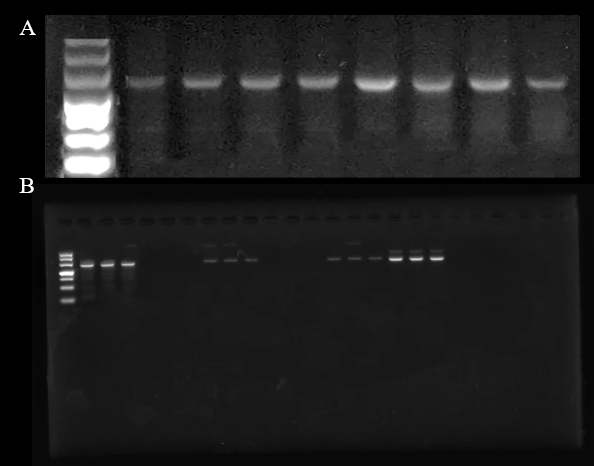


**Figure S5.** Conventional PCR detection of *Puccinia striiformis* f. sp*. tritici* isolates after different days of infection.
